# *trans*-Bis(*N*,*N*-diethyl­ethylenediamine)­nickel(II) dibromide

**DOI:** 10.1107/S1600536810050403

**Published:** 2010-12-08

**Authors:** Skylar J. Ferrara, Joel T. Mague, James P. Donahue

**Affiliations:** a Department of Chemistry, Tulane University, 6400 Freret Street, New Orleans, Louisiana 70118-5698, USA

## Abstract

The structure of the title compound, [Ni(C_6_H_16_N_2_)_2_]Br_2_ or [Ni(Et_2_en)_2_]Br_2_ (Et_2_en is asymmetric *N*,*N*-diethyl­ethylene­diamine), containing an Ni^II^ atom (site symmetry 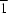) in square-planar NiN_4_ coordination, is described and contrasted with related structures containing Ni^II^ in octa­hedral coordination with axial *X* ^−^ ligands (*X* ^−^ = variable anions). The dialkyl­ated N atom has an appreciably longer bond length to the Ni^II^ atom [1.9666 (13) Å] than does the unsubstituted N atom [1.9202 (14) Å]. The Ni—N bond lengths in [Ni(Et_2_en)_2_]Br_2_ are significantly shorter than corresponding values in tetra­gonally distorted [Ni(Et_2_en)_2_ *X*_2_] compounds (*X* = ^−^O_2_CCF_3_, OH_2_, or ^−^NCS), which have a triplet ground state. The electronic configuration in these axially ligated [Ni(Et_2_en)_2_ *X*_2_] compounds populates the metal-based *d*_x_^2^_-y_^2^ orbital, which is Ni—N anti­bonding in character. Each Et_2_en ligand in each [Ni(Et_2_en)_2_]^2+^ cation forms a pair of N—H⋯Br hydrogen bonds to the Br^−^ anions, one above and below the NiN_4_ square plane. Thus, a ribbon of alternating Br^−^ pairs and [Ni(Et_2_en)_2_]^2+^ cations that are canted at 65° relative to one another is formed by hydrogen bonds.

## Related literature

The synthesis of a broad variety of Ni(Et_2_en)_2_ *X*_2_ compounds is described by Goodgame & Venanzi (1963 ▶). The compounds containing Ni^II^ in octa­hedral coordination with axial *X* ligands have been structurally characterized for *X* = ^−^O_2_CCF_3_ (Senocq *et al.*, 1999 ▶), ^−^NCS (Lever *et al.*, 1983 ▶) and H_2_O with non-coordinated Cl^−^ counter-anions (Ihara *et al.*, 1991 ▶). [Ni(Et_2_en)_2_][ClO_4_]_2_ containing a square-planar centrosymmetric cation has been identified as having triclinic (Ikeda *et al.*, 1995 ▶; Narayanan & Bhadbhade, 1998 ▶) and monoclinic (Hayami *et al.*, 2009 ▶) polymorphs.
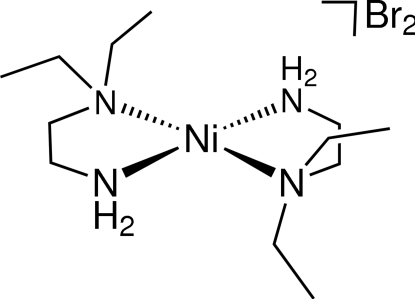


## Experimental

### 

#### Crystal data

[Ni(C_6_H_16_N_2_)_2_]Br_2_
*M* *_r_* = 450.95
Monoclinic, 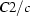

*a* = 12.837 (3) Å
*b* = 11.162 (3) Å
*c* = 13.244 (3) Å
β = 106.543 (4)°
*V* = 1819.2 (8) Å^3^
*Z* = 4
Mo *K*α radiation
μ = 5.45 mm^−1^
*T* = 100 K
0.05 × 0.05 × 0.05 mm

#### Data collection

Bruker APEXI CCD diffractometer
Absorption correction: multi-scan (*SADABS*; Sheldrick, 2008*b* ▶) *T*_min_ = 0.623, *T*_max_ = 0.772
7870 measured reflections
2130 independent reflections
2029 reflections with *I* > 2σ(*I*)
*R*_int_ = 0.024

#### Refinement

*R*[*F* ^2^ > 2σ(*F* ^2^)] = 0.019
*wR*(*F* ^2^) = 0.050
*S* = 1.06
2130 reflections
153 parameters
All H-atom parameters refined
Δρ_max_ = 0.57 e Å^−3^
Δρ_min_ = −0.43 e Å^−3^

### 

Data collection: *APEX2* (Bruker, 2009 ▶); cell refinement: *SAINT* (Bruker, 2008 ▶); data reduction: *SAINT*; program(s) used to solve structure: *SHELXS97* (Sheldrick, 2008*a* ▶); program(s) used to refine structure: *SHELXL97* (Sheldrick, 2008*a* ▶); molecular graphics: *SHELXTL* (Sheldrick, 2008*a* ▶); software used to prepare material for publication: *SHELXTL*.

## Supplementary Material

Crystal structure: contains datablocks I, global. DOI: 10.1107/S1600536810050403/wm2433sup1.cif

Structure factors: contains datablocks I. DOI: 10.1107/S1600536810050403/wm2433Isup2.hkl

Additional supplementary materials:  crystallographic information; 3D view; checkCIF report

## Figures and Tables

**Table 1:** Hydrogen-bond geometry (Å, °)

*D*—H⋯*A*	*D*—H	H⋯*A*	*D*⋯*A*	*D*—H⋯*A*
N2—H2*N*⋯Br1^i^	0.88 (2)	2.47 (2)	3.3524 (15)	176.3 (19)
N2—H1*N*⋯Br2^i^	0.84 (2)	2.64 (2)	3.4381 (15)	157.9 (19)

Symmetry code: (i) 
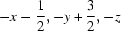
.

**Fig. 1.**
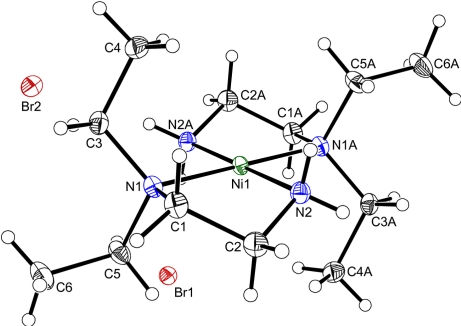
[Ni(Et2en)2]Br2 shown with 50% probability ellipsoids.

**Fig. 2.**
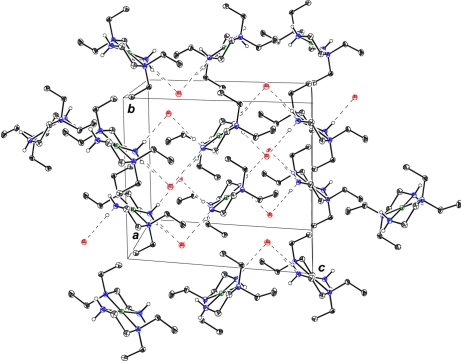
Unit cell packing diagram for [Ni(Et2en)2]Br2 with N—H···Br- hydrogen bonds illustrated. For clarity, all hydrogen atoms other than those involved in hydrogen bonding are removed.

**Fig. 3.**
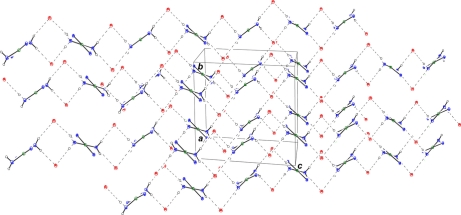
A packing diagram illustrating the one-dimensional ribbon formed by hydrogen bonds between Br- anions and [Ni(Et2en)2]2+ cations. For greater clarity, all carbon atoms in the [Ni(Et2en)2]2+ cations are removed.

## supplementary crystallographic information

### Comment

Complexes of the general type Ni(Et_2_en)_2_*X*_2_ (Et_2_en = asymmetric *N*,*N*-diethylethylenediamine; *X*^-^ = variable anions) were first synthesized by Goodgame & Venanzi (1963) as compounds which, depending on the particular identity of *X*^-^, might reveal triplet and singlet spin states in close enough energetic proximity that a thermal distribution between them could be observed. The compounds with *X* = halide, ^-^O_2_CR, NO_2_^-^ and ^-^NCS were formulated with a tetragonally-distorted octahedral coordination around Ni^II^ with axial *X*^-^ ligands, while those with *X*^-^ = ClO_4_^-^, BF_4_^-^, BPh_4_^-^ and NO_3_^-^ were recognized as being complexes with Ni^II^ in square-planar coordination with noncoordinating *X*^-^ counterions. Apart from a tendency for the F^-^ , Cl^-^ and Br^-^ compounds to adsorb ambient moisture and form the corresponding hydrates, the Ni(Et_2_en)_2_*X*_2_ compounds are readily prepared and handled. The ease with which *X*^-^ is varied and the amenability of the Ni(Et_2_en)_2_*X*_2_ compound set to straightforward magnetic susceptibility and UV-vis spectroscopic measurements, disposes it as a useful vehicle for teaching the spectrochemical series in an undergraduate laboratory context.

In the course of a revised and expanded laboratory experiment with Ni(Et_2_en)_2_*X*_2_ compounds at Tulane University, undergraduate students produced diffraction quality crystal samples of the unhydrated bromide compound by using the vial-in-vial vapor diffusion technique. Following a data collection at 100 K, structure solution and refinement of this bromide compound revealed the Ni^II^ atom to have square-planar coordination in the crystalline state (Scheme 1, Figure 1) rather than octahedral coordination as assumed by Goodgame & Venanzi (1963). The Ni···Br interatomic distances are 4.3048 (6) and 5.0032 (8) Å, which are too large to be compatible with any bonding interaction between them. Despite the noncoordination of Br^-^ in the crystal structure, the possibility of weak axial interaction by Br^-^ with the Ni^II^ atom in solution is not precluded.

The [Ni(Et_2_en)_2_]^2+^ cation resides on an inversion center in *C*2/*c* such that only two independent and appreciably different Ni—N bond lengths occur (1.9202 (14), 1.9666 (13) Å). The longer Ni—N interatomic distance found for the dialkylated nitrogen atom may be plausibly attributed to steric effects exerted by the ethyl groups. Square planar [Ni(Et_2_en)_2_]^2+^ has also been structurally characterized as the perchlorate salt (Ikeda *et al.*, 1995; Narayanan & Bhadbhade, 1998; Hayami *et al.*, 2009) and observed to have similar Ni—N bond lengths of 1.930 (3) and 1.976 (2) Å (Ikeda *et al.*, 1995).

The Ni—N bond lengths found for square-planar [Ni(Et_2_en)_2_]^2+^ contrast with those observed in related structures with axial ligands. The corresponding Ni—N bond lengths are 2.065 (2) and 2.262 (2) Å where *X* = ^-^O_2_CCF_3_ (Senocq *et al.*, 1999), 2.064 (3) and 2.271 (3) Å where *X* = OH_2_ (Ihara *et al.*, 1991), and 2.083 (2) and 2.318 (2) Å (averaged values for two independent molecules) where *X* = ^-^NCS (Lever *et al.*, 1983). The longer Ni—N bond lengths in these latter compounds are due to a triplet electronic configuration in which the *d*_x^2^-y^2^_ orbital is singly occupied. This orbital, which is antibonding in character with respect to metal and ligand, is unoccupied in square-planar [Ni(Et_2_en)_2_]^2+^, thus accounting for the pronounced shortening observed in its Ni—N bond lengths.

Although the Br^-^ ions do not have a bonding interaction with the Ni^II^ atoms in the title crystal structure, they participate in a one-dimensional ribbon of hydrogen bonds, the formation of which is undoubtedly the principal factor governing the pattern of crystal packing. As illustrated in Figure 2, adjacent [Ni(Et_2_en)_2_]^2+^ cations are inclined at an angle of 65° and form a pseudo herringbone (or zigzag) pattern in the plane of the *a* and *c* unit cell axes. Two bromide anions are positioned between adjacent nickel complexes, one above and one below the square-planar complex cations. The pronounced canting of the [Ni(Et_2_en)_2_]^2+^ cations orients each NH_2_ group on each Et_2_en ligand to form two hydrogen bonds, one above and one below the square plane. Thus, each [Ni(Et_2_en)_2_]^2+^ cation forms four hydrogen bonds, two above and two below the square plane at opposite ends of the nickel complex cation. Approximate squares of hydrogen bonds are formed, with nitrogen atoms and bromide anions on opposing vertices and sides ~3.4 Å in length . Figure 3 presents an alternative rendering of this hydrogen bonding pattern with all the carbon atoms of the Et_2_en ligands removed for clarity.

### Experimental

Orange diamondoid crystals of [Ni(Et_2_en)_2_]Br_2_ grew by diffusion of *^t^*BuOMe vapor into a dry methanol solution in a sealed vial. The MeOH solution was prepared by stirring an excess of powdered [Ni(Et_2_en)_2_]Br_2_ in several mL of dry MeOH for a period of five minutes. This heterogeneous mixture was then passed though a pad of packed Celite to remove all undissolved material and produce a homogeneous filtrate.

### Refinement

H-atoms were identified in the final electron density map. Their positions were refined with isotropic thermal parameters.

### Crystal data

**Table:**

[Ni(C_6_H_16_N_2_)_2_]Br_2_	*F*(000) = 920
*M**_r_* = 450.95	*D*_x_ = 1.647 Mg m^−^^3^
Monoclinic, *C*2/*c*	Mo *K*α radiation, λ = 0.71073 Å
Hall symbol: -C 2yc	Cell parameters from 6699 reflections
*a* = 12.837 (3) Å	θ = 2.5–28.3°
*b* = 11.162 (3) Å	µ = 5.45 mm^−^^1^
*c* = 13.244 (3) Å	*T* = 100 K
β = 106.543 (4)°	Diamondoid, orange
*V* = 1819.2 (8) Å^3^	0.05 × 0.05 × 0.05 mm
*Z* = 4	

### Data collection

**Table:**

Bruker APEXI CCD diffractometer	2130 independent reflections
Radiation source: fine-focus sealed tube	2029 reflections with *I* > 2σ(*I*)
graphite	*R*_int_ = 0.024
φ and ω scans	θ_max_ = 28.3°, θ_min_ = 2.5°
Absorption correction: multi-scan (*SADABS*; Sheldrick, 2008*b*)	*h* = −17→16
*T*_min_ = 0.623, *T*_max_ = 0.772	*k* = −14→14
7870 measured reflections	*l* = −17→17

### Refinement

**Table:**

Refinement on *F*^2^	Primary atom site location: structure-invariant direct methods
Least-squares matrix: full	Secondary atom site location: difference Fourier map
*R*[*F*^2^ > 2σ(*F*^2^)] = 0.019	Hydrogen site location: difference Fourier map
*wR*(*F*^2^) = 0.050	All H-atom parameters refined
*S* = 1.06	*w* = 1/[σ^2^(*F*_o_^2^) + (0.0311*P*)^2^ + 1.2408*P*] where *P* = (*F*_o_^2^ + 2*F*_c_^2^)/3
2130 reflections	(Δ/σ)_max_ = 0.001
153 parameters	Δρ_max_ = 0.57 e Å^−^^3^
0 restraints	Δρ_min_ = −0.43 e Å^−^^3^

### Special details

**Table:**

Experimental. The diffraction data were collected in three sets of 606 frames (0.3 deg. width in ω at φ = 0, 120 and 240 deg. A scan time of 10 sec/frame was used.
Geometry. All esds (except the esd in the dihedral angle between two l.s. planes) are estimated using the full covariance matrix. The cell esds are taken into account individually in the estimation of esds in distances, angles and torsion angles; correlations between esds in cell parameters are only used when they are defined by crystal symmetry. An approximate (isotropic) treatment of cell esds is used for estimating esds involving l.s. planes.
Refinement. Refinement of *F*^2^ against ALL reflections. The weighted R-factor wR and goodness of fit S are based on *F*^2^, conventional R-factors R are based on *F*, with *F* set to zero for negative *F*^2^. The threshold expression of *F*^2^ > 2σ(*F*^2^) is used only for calculating R-factors(gt) etc. and is not relevant to the choice of reflections for refinement. R-factors based on *F*^2^ are statistically about twice as large as those based on *F*, and R- factors based on ALL data will be even larger.

### Fractional atomic coordinates and isotropic or equivalent isotropic displacement parameters (Å^2^)

**Table:**

	*x*	*y*	*z*	*U*_iso_*/*U*_eq_	
Br1	0.0000	0.913257 (18)	0.2500	0.01466 (7)	
Br2	0.0000	0.469242 (19)	0.2500	0.01838 (7)	
Ni1	−0.2500	0.7500	0.0000	0.01079 (7)	
N1	−0.31881 (11)	0.67603 (11)	0.09965 (10)	0.0131 (3)	
N2	−0.39293 (11)	0.80349 (12)	−0.07621 (11)	0.0141 (3)	
C1	−0.43989 (13)	0.69026 (14)	0.05604 (13)	0.0163 (3)	
C2	−0.46277 (14)	0.80667 (14)	−0.00423 (13)	0.0178 (3)	
C3	−0.29344 (13)	0.54467 (14)	0.11697 (12)	0.0160 (3)	
C4	−0.29416 (14)	0.48022 (15)	0.01586 (13)	0.0189 (3)	
C5	−0.27917 (14)	0.74426 (15)	0.20169 (12)	0.0183 (3)	
C6	−0.31232 (17)	0.69197 (17)	0.29384 (14)	0.0238 (4)	
H1N	−0.4012 (17)	0.868 (2)	−0.1105 (17)	0.020 (5)*	
H2N	−0.4197 (18)	0.748 (2)	−0.1241 (17)	0.023 (5)*	
H1A	−0.4657 (16)	0.6263 (19)	0.0066 (16)	0.015 (5)*	
H1B	−0.4752 (18)	0.6886 (17)	0.1114 (17)	0.018 (5)*	
H2A	−0.538 (2)	0.811 (2)	−0.045 (2)	0.030 (6)*	
H2B	−0.4442 (17)	0.8744 (19)	0.0408 (16)	0.017 (5)*	
H3A	−0.2203 (16)	0.5385 (17)	0.1685 (14)	0.010 (4)*	
H3B	−0.3443 (15)	0.5112 (18)	0.1468 (14)	0.012 (4)*	
H4A	−0.231 (2)	0.498 (2)	−0.0054 (17)	0.037 (6)*	
H4B	−0.293 (2)	0.394 (2)	0.033 (2)	0.037 (6)*	
H4C	−0.3579 (19)	0.492 (2)	−0.0417 (18)	0.031 (6)*	
H5A	−0.2023 (19)	0.746 (2)	0.2170 (17)	0.023 (5)*	
H5B	−0.3036 (18)	0.8251 (19)	0.1900 (17)	0.018 (5)*	
H6A	−0.291 (2)	0.744 (2)	0.349 (2)	0.036 (6)*	
H6B	−0.388 (3)	0.680 (3)	0.281 (2)	0.052 (9)*	
H6C	−0.280 (2)	0.615 (3)	0.313 (2)	0.040 (7)*	

### Atomic displacement parameters (Å^2^)

**Table:**

	*U*^11^	*U*^22^	*U*^33^	*U*^12^	*U*^13^	*U*^23^
Br1	0.01334 (11)	0.01570 (11)	0.01413 (11)	0.000	0.00262 (8)	0.000
Br2	0.01588 (12)	0.01737 (12)	0.01981 (12)	0.000	0.00175 (8)	0.000
Ni1	0.00960 (13)	0.01277 (13)	0.01037 (13)	−0.00131 (9)	0.00344 (9)	0.00080 (9)
N1	0.0117 (6)	0.0162 (6)	0.0112 (6)	−0.0021 (5)	0.0029 (5)	0.0002 (5)
N2	0.0135 (6)	0.0150 (6)	0.0141 (6)	−0.0002 (5)	0.0042 (5)	0.0013 (5)
C1	0.0110 (7)	0.0213 (8)	0.0174 (7)	−0.0017 (6)	0.0052 (6)	0.0013 (6)
C2	0.0139 (8)	0.0218 (8)	0.0196 (8)	0.0024 (6)	0.0075 (6)	0.0015 (6)
C3	0.0172 (7)	0.0159 (7)	0.0148 (7)	−0.0021 (6)	0.0043 (6)	0.0022 (6)
C4	0.0207 (8)	0.0157 (7)	0.0197 (8)	−0.0023 (6)	0.0051 (6)	−0.0012 (6)
C5	0.0211 (8)	0.0211 (8)	0.0132 (7)	−0.0071 (6)	0.0058 (6)	−0.0032 (6)
C6	0.0303 (10)	0.0288 (9)	0.0149 (8)	−0.0087 (7)	0.0105 (7)	−0.0026 (7)

### Geometric parameters (Å, °)

**Table:**

Ni1—N2^i^	1.9202 (14)	C2—H2B	0.95 (2)
Ni1—N2	1.9202 (14)	C3—C4	1.518 (2)
Ni1—N1	1.9666 (13)	C3—H3A	0.993 (19)
Ni1—N1^i^	1.9666 (13)	C3—H3B	0.933 (19)
N1—C1	1.505 (2)	C4—H4A	0.96 (2)
N1—C3	1.505 (2)	C4—H4B	0.99 (3)
N1—C5	1.508 (2)	C4—H4C	0.96 (2)
N2—C2	1.483 (2)	C5—C6	1.519 (2)
N2—H1N	0.84 (2)	C5—H5A	0.95 (2)
N2—H2N	0.88 (2)	C5—H5B	0.95 (2)
C1—C2	1.509 (2)	C6—H6A	0.92 (3)
C1—H1A	0.96 (2)	C6—H6B	0.95 (3)
C1—H1B	0.97 (2)	C6—H6C	0.96 (3)
C2—H2A	0.96 (3)		
			
N2^i^—Ni1—N2	180.0	N2—C2—H2B	109.5 (12)
N2^i^—Ni1—N1	93.51 (6)	C1—C2—H2B	112.1 (12)
N2—Ni1—N1	86.49 (6)	H2A—C2—H2B	110.1 (18)
N2^i^—Ni1—N1^i^	86.49 (6)	N1—C3—C4	112.36 (13)
N2—Ni1—N1^i^	93.51 (6)	N1—C3—H3A	107.0 (11)
N1—Ni1—N1^i^	180.00 (7)	C4—C3—H3A	109.9 (11)
C1—N1—C3	108.44 (11)	N1—C3—H3B	107.9 (12)
C1—N1—C5	109.81 (12)	C4—C3—H3B	110.8 (12)
C3—N1—C5	110.62 (12)	H3A—C3—H3B	108.7 (15)
C1—N1—Ni1	108.08 (9)	C3—C4—H4A	111.6 (14)
C3—N1—Ni1	113.09 (10)	C3—C4—H4B	104.9 (14)
C5—N1—Ni1	106.74 (9)	H4A—C4—H4B	109 (2)
C2—N2—Ni1	109.36 (10)	C3—C4—H4C	115.2 (14)
C2—N2—H1N	108.6 (14)	H4A—C4—H4C	110.1 (19)
Ni1—N2—H1N	120.3 (15)	H4B—C4—H4C	106 (2)
C2—N2—H2N	107.2 (14)	N1—C5—C6	115.20 (13)
Ni1—N2—H2N	106.2 (14)	N1—C5—H5A	105.6 (14)
H1N—N2—H2N	104 (2)	C6—C5—H5A	110.5 (13)
N1—C1—C2	108.55 (12)	N1—C5—H5B	108.7 (13)
N1—C1—H1A	107.5 (12)	C6—C5—H5B	109.8 (13)
C2—C1—H1A	107.6 (12)	H5A—C5—H5B	106.6 (19)
N1—C1—H1B	111.3 (13)	C5—C6—H6A	108.2 (16)
C2—C1—H1B	110.8 (12)	C5—C6—H6B	114.6 (19)
H1A—C1—H1B	110.9 (17)	H6A—C6—H6B	106 (2)
N2—C2—C1	104.89 (13)	C5—C6—H6C	111.4 (16)
N2—C2—H2A	109.1 (15)	H6A—C6—H6C	111 (2)
C1—C2—H2A	111.1 (14)	H6B—C6—H6C	105 (2)

Symmetry codes: (i) −*x*−1/2, −*y*+3/2, −*z*.

### Hydrogen-bond geometry (Å, °)

**Table:**

*D*—H···*A*	*D*—H	H···*A*	*D*···*A*	*D*—H···*A*
N2—H2N···Br1^i^	0.88 (2)	2.47 (2)	3.3524 (15)	176.3 (19)
N2—H1N···Br2^i^	0.84 (2)	2.64 (2)	3.4381 (15)	157.9 (19)

Symmetry codes: (i) −*x*−1/2, −*y*+3/2, −*z*.
